# Sustainable biosynthesis of valuable diterpenes in microbes

**DOI:** 10.1016/j.engmic.2022.100058

**Published:** 2022-11-10

**Authors:** Yanbin Liu, Xixian Chen, Congqiang Zhang

**Affiliations:** Singapore Institute of Food and Biotechnology Innovation (SIFBI), Agency for Science Technology and Research (A*STAR), 31 Biopolis Way, Level 6 Nanos building, Singapore 138669, Singapore

**Keywords:** Diterpene/ diterpenoid, Secondary metabolites, Synthetic biology, Metabolic engineering, Biosynthesis

## Abstract

Diterpenes, or diterpenoids, are the most abundant and diverse subgroup of terpenoids, the largest family of secondary metabolites. Most diterpenes possess broad biological activities including anti-inflammatory, antiviral, anti-tumoral, antimicrobial, anticancer, antifungal, antidiabetic, cardiovascular protective, and phytohormone activities. As such, diterpenes have wide applications in medicine (e.g., the anticancer drug Taxol and the antibiotic pleuromutilin), agriculture (especially as phytohormones such as gibberellins), personal care (e.g., the fragrance sclareol) and food (e.g., steviol glucosides as low-calorie sweeteners) industries. Diterpenes are biosynthesized in a common route with various diterpene synthases and decoration enzymes like cytochrome P450 oxidases, glycosidases, and acyltransferases. Recent advances in DNA sequencing and synthesis, omics analysis, synthetic biology, and metabolic engineering have enabled efficient production of diterpenes in several chassis hosts like *Escherichia coli, Saccharomyces cerevisiae, Yarrowia lipolytica, Rhodosporidium toruloides*, and *Fusarium fujikuroi*. This review summarizes the recently discovered diterpenes, their related enzymes and biosynthetic pathways, particularly highlighting the microbial synthesis of high-value diterpenes directly from inexpensive carbon sources (e.g., sugars). The high titers (>4 g/L) achieved mean that some of these endeavors are reaching or close to commercialization. As such, we envisage a bright future in translating microbial synthesis of diterpenes into commercialization.

## Graphical abstract

**Figure:**
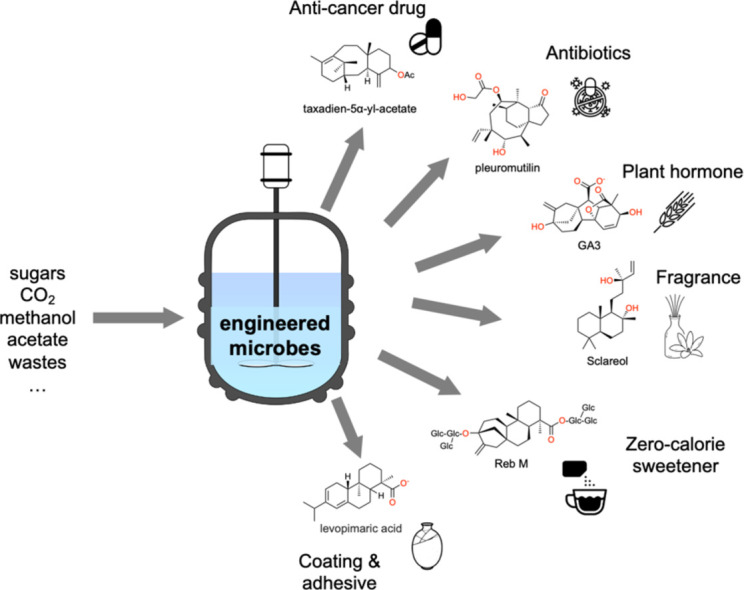

## 1 Natural diterpenes

According to a strict chemical definition, terpenes are molecules without oxygen and terpenoids are the same molecules but with oxygen. However, researchers often use these two terms interchangeably. Here, we use “terpenes” to represent both terpenes and terpenoids with or without oxygen. Similarly, we use “diterpenes” to cover both diterpenes and diterpenoids.

**Fig. 1:**
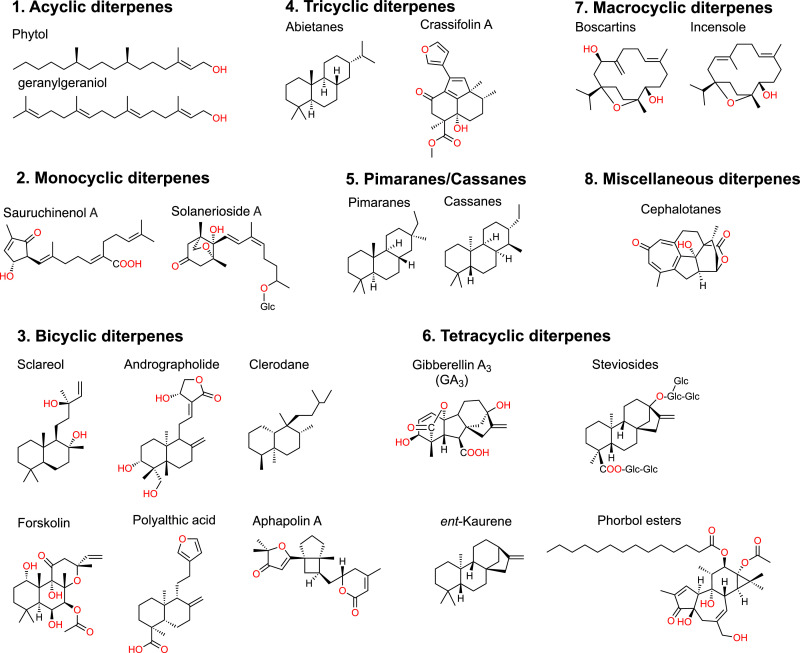
Structure of the representative diterpenes of various rings.

To date, >90,000 terpenes have been found in nature [1], being especially prevalent in plants, fungi, bacteria, and marine invertebrates. Diterpenes are a subclass of terpenes containing 20 carbons (C20) with wide applications in medicine, agriculture, personal care, and food industry. Diterpenes are structurally diverse with 4 C_5_-isoprene units joining from head to tail; they are naturally found in various organisms including plants, marine microorganisms, sponges, insects, and fungal species [2], [3], [4], [5]. Diterpenes can be structurally classified by the number of rings, including acyclic, monocyclic, bicyclic, tricyclic, tetracyclic, macrocyclic, and other miscellaneous structures [6]. Some representative structures are shown in Fig. 1. Diterpenes possess numerous biological activities including anti-inflammatory, antiviral, phytohormone, anti-tumoral, antimicrobial, anticancer, antifungal, antidiabetic, and cardiovascular protective activities (see review [7]). Table 1 lists selected examples of naturally produced diterpenes and their biological activities.

(1) acyclic, (2) monocyclic, (3) bicyclic, (4) tricyclic, (5) pimaranes/cassanes, (6) tetracyclic, (7) macrocyclic and (8) miscellaneous.

**Table 1:** Natural diterpenoids.

**Name**	Major source	Bioactivities	Refs.
**1. Acyclic diterpenes**			
Phytane, Phytol	Plants, alga and cyanabacteria	antioxidant, anti-inflammatory	[11,156]
Geranylgeraniol, tetrahydrogeranylgeraniol, dihydrogeranylgeraniol	Plants	lipid mediators	[157]
**2. Monocyclic diterpenes**			
Sauruchinenols	*Saururus chinensis*	anti-inflammatory	[13]
Solaneriosides	*Solanum erianthum*	anti-inflammatory	[14]
Retinol	Plants, alga and microbes	antioxidant, anti-inflammatory, Vitamin A	[158], [159], [160]
Retinal	Plants, alga and microbes	antioxidant, anti-inflammatory, Vitamin A	[160]
Retinoic acid	Plants, alga and microbes	antioxidant, anti-inflammatory, Vitamin A	[160,161]
**3. Bicyclic diterpenes**			
Aphapolins	*Aphanamixis polystachya*	anti-inflammatory	[12]
Leojapones	*Leonurus japonicu*	anti-platelet aggregation	[17]
Loxocalyxins	*Loxocalyx urticifolius*	anti-cancer	[19]
Cinereanoids	*Roylea cinerea*	anti-tumor	[20]
Norlabdane	*Salvia sahendica*	anti-inflammatory	[21]
Sclareol	*Salvia sclarea*	antifungal, anti-inflammatory	[58,162,163]
Tetranorlabdanes	*Aspergillus wentii, Oidiodendron truncate, Maytenus hookeri, Elettaria cardamomum*	anti-inflammatory	[27,[164], [165], [166]]
Galangalditerpenes	*Alpinia galanga, Curcuma amada*	anti-melanogenesis	[28,29]
Microtropiosides	*Microtropis japonica*	anti-tumor, antiviral	[33]
Phocantosides	*Pholidota cantonensis*	anti-cancer	[34]
Forskolins	*Coleus forskohlii*	anti-cancer	[167]
Andrographolides	*Andrographis paniculata*	anti-cancer	[168,169]
Clerodane	Various plants, fungi, bacteria and marine sponges	insect antifeedant	[36,[172], [173], [174]]
**4. Tricyclic diterpenes**			
Abietanes	Various plants and certain fungi	antiviral, anti-tumor	[38]
Carnosic acid	*Rosmarinus officinalis*	anti-inflammatory, anti-tumor	[132,140]
Levopimaric acid	Conifers	antibacterial, cardiovascular	[142]
Sahandone	*Salvia chloroleuca*	antifungal	[175]
Cyathane	*Various basidiomycetous fungi*	anti-inflammatory, anti-tumor	[176]
Commiphoranes	*Resina Commiphora*	anti-inflammatory	[177,178]
Crassifolins	*Croton crassifolius*	anti-tumor, anti-cancer	[170,171]
Jatromulone	*Jatropha multifida*	anti-cancer	[179,180]
Pleuromutilin	*Pleurotus mutilis, Pleurotus passeckerianus*	antibiotics, antimicrobial	[122,181]
Sageone	*Salvia apiana*	opioid receptor binding	[182]
Salmitiorins	*Salvia miltiorrhiza*	anti-inflammatory	[183,184]
Ganxincastanic acids	*Salvia przewalskii*	anti-cancer	[185]
Salprzelactones	*Salvia przewalskii*	antibiotic	[186]
Plebeins	*Salvia plebeian*	anti-cancer	[187]
Sideritins	*Sideritis montana*	anti-cancer	[188]
Teydeadione	*Nepeta teydea*	anti-cancer, insect antifeedant	[189]
Serrins	*Isodon serra*	anti-inflammatory, anti-cancer	[190]
Tripterycosides	*Tripterygium wilfordii*	anti-inflammatory	[191]
Taxodikaloids	*Taxodium ascendens*	neuroprotective	[192]
Eupractenoids	*Euphorbia ebracteolata*	antidiabetic	[193]
Fischeriabietanes	*Euphorbia fischeriana*	antibiotic, anti-inflammatory	[194]
Crotolaevigatones	*Croton laevigatus*	anti-cancer	[195]
Trigonoreidons	*Kaempferia roscoeana*	antituberculosis	[196]
Roscotanes/Roscoranes	*Kaempferia roscoeana*	antimalarial	[197]
Nepetaefolins	*Caryopteris nepetaefolia*	anti-cancer	[198]
Kwangpenes	*Callicarpa kwangtungensis*	anti-inflammatory	[199]
Callicapoic acids	*Callicarpa macrophylla*	anti-inflammatory	[200]
**5. Pimaranes, cassanes and related diterpenes**			
Callicarpenes	*Callicarpa macrophylla*	anti-inflammatory	[201]
Sphaeropsidins	*Aspergillus candidus*	anti-cancer	[202]
Botrysphins	*Botrysphaeria laricina*	anti-cancer	[203]
Isopimarane	*Lyonia ovalifolia*	antimalarial	[204]
Strobols	*Siegesbeckia pubescens*	anti-cancer	[205]
Siegesides	*Siegesbeckia pubescens*	anti-cancer	[206]
Euonymusisopimaric acids	*Euonymus oblongifolius*	anti-inflammatory	[207]
Libertellenones	*Apiospora montagnei*	anti-cancer	[208]
Euphominoids	*Euphorbia milii*	antiviral	[209]
Rosololactone	*Trichothecium roseum*	anti-cancer	[210]
Tagalenes	*Ceriops tagal*	antimalarial	[211]
Icacinlactones	plant and fungi	agriculture and medicine	[4,40]
Erythrofordins	*Erythrophleum fordii*	antiviral, anti-cancer	[212]
Erythroformines	*Erythrophleum fordii*	antimalarial, antidiabetic	[213]
Furanocassane sucupiranins	*Bowdichia virgilioides*	antiviral, anti-cancer	[4,214,215]
Caesmimosins	*Caesalpinia mimosoides*	anti-cancer	[216]
Caesalppans	*Caesalpinia sappan*	anti-inflammatory, antimalarial	[217]
Caesalsappanins	*Caesalpinia sappan*	antimalarial	[218]
Cleistanthane	*Trigonostemon howii*	anti-inflammatory	[219]
Phyllanes	*Phyllanthus acidus*	anti-cancer	[220]
Neocyathins	*Cyathus africanus*	anti-inflammatory	[221]
Erinacines	*Hericium erinaceus*	anti-neurodegenerative diseases, anti-inflammatory	[143,144]
**6. Tetracyclic diterpenes**			
GA_1_, GA_3_, GA_4_, GA_7_	Plants and *Fusarium fujikuroi*	anti-cancer, plant promoting	[114,222]
Beyerenoic/kaurenoic acids	*Perymenium buphthalmoides*	anti-plant disease	[223]
Spriograterpene	*Penicillium granulatum*	spiro-tetracyclic	[245]
Phorbol esters	*Croton tiglium, Aquilaria malaccensis*	anti-cancer, anti-inflammatory	[249,250]
Steviosides	*Stevia rebaudiana*	sweeteners	[224]
Steviolbiosides	*Stevia rebaudiana*	sweeteners	[46]
Rebaudiosides	*Stevia rebaudiana*	sweeteners	[46,225]
Cafestol/kahweol	Coffee	anti-tumor, anti-inflammatory	[226], [227], [228]
*ent*-kaur-2-one-16β,17-diol	*Rubus corchorifolius*	anti-cancer	[229]
*ent*-3-oxokaurane-16β,17-diol	*Euphorbia fischeriana*	anti-tumor	[230]
ent-Kaurane-16β,17,19-triol-3-one	*Euphorbia stracheyi*	anti-cancer	[231]
Decorenes	*Gochnatia decora*	anti-inflammatory	[232]
Leontocins	*Leontopodium leontopodioides*	anti-inflammatory	[233]
*seco*-Grayananes	*Pieris japonica, Pieris formosa*	antifeedant	[234, 235]
Aromaticanes	*Curcuma aromatica*	antioxidant	[236]
Aleuritopsis	*Aleuritopteris argentea*	anti-tumor	[237]
Inflatins	*Tolypocladium inflatum*	anti-cancer	[238]
Harziene	*Trichoderma species*	antiviral	[239]
**7. Macrocyclic diterpenes**			
Deheiculatins	*Macaranga deheiculata*	antidiabetic	[240]
Launines	*Croton laui*	anti-tumor	[241]
Taxol/Taxanes	*Taxus wallichiana*	anti-tumor, antidiabetic	[242], [243], [244]
Gaditanones	*Euphorbia gaditana*	activate PKCs	[246]
Euphoractanes	*Euphorbia species*	anti-cancer	[50,247]
Crotodichogamoins	*Croton dichogamus*	anti-cancer	[248]
Daphnanes	*Trigonostemon sp.*	anti-inflammatory	[251,252]
Jolkinoates	*Euphorbia piscatoria*	anti-cancer	[253]
Vibsane	*Viburnum sp.*	anti-cancer	[254]
**8. Miscellaneous diterpenes**			
Damasterpenes	*Nigella damascene*	spice	[54]
14-hydroxycyclooctatin	*Streptomyces violascens*	antiviral	[55]
20-oxohainanolidol	*Cephalotaxus sinensis*	antiviral	[255]
Cephanolides	*Cephalotaxus sinensis*	anti-tumor	[56]
Vulgarisins	*Prunella vulgaris*	anti-tumor	[57]
Phomactins	*Phoma sp., Ishige okumare, Briatrispora sp.*	anti-tumor	[3,256]
Harzian(di)one	*Trichoderma species*	antimalarial	[257]
Cinnamomols	*Cinnamomum cassia*	immunostimulative	[258]

Acyclic diterpenes are less frequently found in nature. However, they are critical for research on other diterpenes [8]. Phytane (C_20_H_42_) is an isoprenoid alkane formed when phytol (C_20_H_40_O), a constituent of chlorophyll and a precursor of vitamin E and K1, loses its hydroxyl group [9,10]. Phytol and some of its derivatives are the most prevalent acyclic diterpenes, exerting a wide range of biological effects, including anxiolytic, metabolism-modulating, cytotoxic, antioxidant, autophagy- and apoptosis-inducing, antinociceptive, anti-inflammatory, immune-modulating, and antimicrobial effects (see review [11]). Similarly, monocyclic diterpenes such as aphapolins, sauruchinenols and solaneriosides are rare in nature, and known by their anti-inflammatory effects [12], [13], [14].

Bicyclic diterpenes share a structure of two rings in their carbon skeletons, and contain three subgroup members, labdanes, halimanes, and clerodanes. Labdane-related diterpenes (LRDs) are a large group of known bicyclic diterpenes, including over 10% of all terpenoids [15]. LRDs have been widely used in the perfume and food industries for centuries; most display antibacterial, antifungal, antimutagenic, cytotoxic, cytostatic, and anti-inflammatory effects (see review [16]). LRDs and their derivatives like labdane glycosides, are generally isolated from *Lamiaceae* (mint) [17], [18], [19], [20], [21], [22], [23], [24], [25], [26] and *Zingiberaceae* (ginger) [27], [28], [29], [30], [31], [32], [33], [34] (Table 1 and Fig. 1). Forskolin, sclareol, andrographolide, and polyalthic acid are representative LRDs used in research [16,35]. Clerodane-related diterpenes are a widespread class of secondary metabolites isolated from various plants, fungi, bacteria, and marine sponges. They display specialized functions such as antifeedant properties and opioid receptor probes [36]. Halimane-related diterpenes contain a small group of natural products structurally classified between labdane and clerodane diterpenoids [37].

Tricyclic diterpenes structurally contain three rings in their backbone, including abietanes, pimaranes, and cassanes. Abietanes have been isolated from a wide range of plants, especially conifer resins and some fungi, and are interesting for the synthetic, medicinal, and pharmacological communities [4,38]. Pimaranes are biosynthetically formed from the subsequent conversion of geranylgeranyl pyrophosphate (GGPP) into (+)-copalyl and sandaracopimarenyl cations [39]. Pimaranes are frequently produced by plant and fungi, having biological activities with potential applications in agriculture and medicine (see review [40]). Isolated from *Caeselpinia* species, cassanes contain a furan ring or an α, β-butenolide moiety in their diterpene backbone, and display antiviral, anticancer, anti-inflammatory, antimalarial, and antiproliferative activities [4,6].

Tetracyclic diterpenes are widely distributed and biosynthesized by cyclization of suitably oriented tricyclic diterpenes [41]. The most famous tetracyclic diterpenes are gibberellins, which possess the tetracyclic *ent*-gibberellane carbon skeletal structure, arranged in either four or five ring systems where the variable fifth ring is a lactone (see review [42]; Table 1 and Fig. 1). Gibberellins can be classified into two types based on the total carbon number, C_20_-GAs and C_19_-GAs. C_19_-GAs are acidic and their polarity varies according to the number of hydroxyl and carboxyl groups, the saturation level, and the presence of methylene or sugar residues [43]. All bioactive GAs, such as GA_1_, GA_3_, GA_4_ and GA_7_, belong to the C_19_-GAs group [44,45]. Other rich tetracyclic diterpenes are sweetener compounds, diterpene glycosides, isolated from various *Stevia* species [46]. Phorbol esters are other important diol-consisting tetracyclic diterpenes, considered key compounds for drug discovery due to the versatile roles of their receptor, protein kinase C [47,48].

Macrocyclic diterpenes are a group of complex and polycyclic diterpenes with varied and usually highly oxygenated skeletons (Table 1 and Fig. 1). These compounds are generally isolated from *Euphorbia* family plants and exhibit therapeutically relevant biological properties such as antitumor, antiviral, anti-inflammatory, cytotoxic, and multidrug-resistance-reversing activities [49], [50], [51]. To date, a new group of macrocyclic diterpenes, cembrans and cembranoids, gained increasing attention for their anti-human immunodeficiency virus, anticancer, and anti-inflammatory activities [52,53].

To date, numerous novel diterpenes have been discovered and clustered as miscellaneous diterpenes. For example, the spice compound damasterpenes from *Nigella damascene* [54], the fusicoccane skeleton-based 14-hydroxycyclooctatin from *Streptomyces violascens* [55], new cephalotanes from *Cephalotaxus sinensis* [56], and novel skeleton-based vulgarisins from *Prunella vulgaris* [57]. These diterpenes are known due to their significant antibacterial and antiviral activities [58,59].

## 2 Biosynthesis and regulation

To date, the biosynthetic pathways of diterpenes have been elucidated in various organisms (see reviews [58,60,61]). Generally, diterpenes are biosynthesized in three common steps: (1) formation of a shared precursor, (*E, E, E*)-geranylgeranyl diphosphate (GGPP, C_20_) catalyzed by GGPP synthase (GGPPS); (2) construction of the carbon backbones of diterpenes catalyzed by diterpene synthases (DTSs); and (3) diterpene-specific modifications catalyzed by cytochrome P450 oxygenases (CYPs or P450s) and/or other decoration enzymes, *e.g.* glycosyltransferases (GTs) and acyltransferases (ACTs) [60].

GGPP biosynthesis has two major pathways, the mevalonate (MVA) and the methylerythritol 4-phosphate (MEP) pathways. The former uses acetyl-CoA to produce isopentenyl pyrophosphate (IPP) and its isomer dimethylallyl diphosphate (DMAPP), the two precursors to GGPP [62,63]. In contrast, the MEP pathway produces IPP and DMAPP from pyruvate and glyceraldehyde-3-phosphate. The MVA pathway is used in nearly all eukaryotes, archaea, and some gram-positive bacteria while the MEP pathway is present in most gram-negative bacteria, green algae, and the plastids of plants and cyanobacteria (see review [64]). In the MVA pathway, the 3-hydroxy-3-methylglutaryl-CoA reductase (HMG-CoA reductase, HMGR) is the main regulatory target of its downstream products at transcriptional, post-transcriptional, translational, or post-translational levels [65], [66], [67]. Compared to the MVA pathway, the regulation of the MEP pathway is poorly understood [68]. However, the first two enzymes, 1-deoxy-D-xylulose-5-phospate synthase (DXS) and 1-deoxy-D-xylulose-5-phosphate reductoisomerase (DXR), are widely accepted as candidate engineering targets [69], [70], [71], [72].

The carbon skeleton of diterpenes and side-chain modifications are catalyzed by prenyltransferases (PTs) and DTSs. PTs regulate the prenyl carbon chain length while DTSs introduce structural complexity to the molecular scaffolds [73], [74], [75], [76], [77]. Here, we review mainly the roles of DTSs, CYPs, and decoration enzymes in the biosynthesis of diterpenoids.

### 2.1 DTSs

**Fig. 2:**
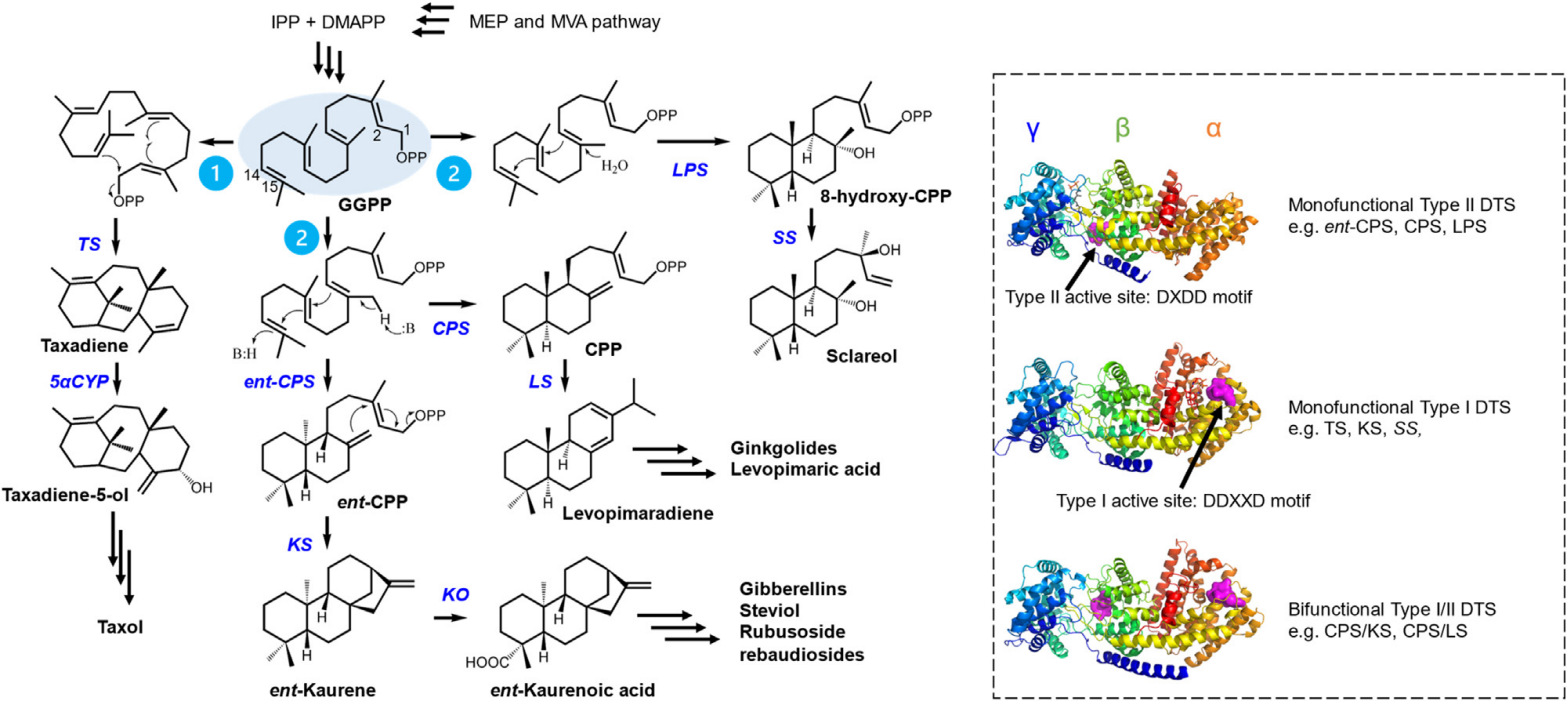
Schematic representation of selected diterpenoid biosynthesis and three DTS types.

DTSs are the gateway enzymes that convert GGPP to a diverse range of hydrocarbon scaffolds. Structurally, most DTSs adopt the α-, β- and γ-domain conformation with a few αβ or α domain only (Fig. 2) [78]. They can be classified into monofunctional Type I DTSs, monofunctional Type II DTSs, and bifunctional Type I/II DTSs. Type I DTS contains the DDXXD motif in its active site, located at the α-domain at the C-terminal, whereas Type II DTS contains the DXDD motif at the interface between the γ- and β-domain near the N-terminal (Fig. 2) [2,78]. Type I DTS initiates the reaction by metal-dependent ionization of the diphosphate group, generating carbocation at the C_1_ position of GGPP (pathway 1, Fig. 2). Taxadiene synthase (TaS) is a typical Type I DTS that catalyzes cyclization between the C_1_ and C_15_ bond after diphosphate loss. Quantum mechanics/molecular mechanics research has identified that this cyclization step has a high energy barrier which limits its activity [79]. Domain truncation to remove γ-domain and γβ-domain of TS from *Taxus brevifolia* (TbTS) resulted in 2750-fold reduction in catalytic efficiency and loss of taxadiene production, respectively [80]. This observation indicates the importance of the β- and γ-domains even if they do not directly participate in catalysis. Type II DTS, on the other hand, initiates the formation of carbocation on the other end of GGPP by protonating the C_15_ position while leaving the diphosphate intact (pathway 2, Fig. 2). The bicyclic hydrocarbon backbone of this diphosphate intermediate (copalyl/labdadienyl diphosphate (CPP or LPP)) is named labdane and the resulting diterpenes are thus named labdane-related diterpenoids [2]. CPP is subsequently transferred to another Type I DTS for further structural diversification. Labdane-related diterpenoids constitute most diterpenes. For example, GAs, the prevalent plant hormones, are a labdane-related diterpenoid. Their precursor, *ent*-kaurene, is mainly biosynthesized through the sequential actions of the monofunctional Type II *ent*-copalyl diphosphate synthase (*ent*-CPS) followed by the monofunctional Type I *ent*-kaurene synthase (KS) in bacteria and higher plants [81]. Based on the crystal structure of *ent*-CPS from *Arabidopsis thaliana* (AtCPS, PDB ID: 4LIX) complexed with a CPP analogue, the second aspartic acid in the DXDD motif functions as the general acid for protonation [82]. Importantly, the magnesium ion in the active site is not for catalysis but for substrate binding [82]. In fungi and early land plants, however, a bifunctional Type I/II DTS (CPS/KS) catalyzes the two-step *ent*-kaurene biosynthesis [83]. Interestingly, the bifunctional CPS/KS has a similar length to monofunctional CPS or KS. Accordingly, gibberellin biosynthesis may have started by fusion of CPS and KS in bacteria and evolved through gene duplication to form two distinct functional enzymes in higher plants [84]. For biosynthesis of labdane-related diterpenoids in heterologous hosts, bifunctional Type I/II DTS might be advantageous due to the proximity of the two active sites to facilitate substrate channeling without the need for another enzyme, thus saving energy and other resources. Interestingly, some Type I and Type II DTSs can introduce water during catalysis and produce diterpene alcohols. For example, the important Ambrox® precursors, sclareol and abienol, are biosynthesized via the intermediate 8-hydroxy-CPP, formed with water at the C-8 position of GGPP (Fig. 2) [85]. A mutagenesis study showed that the Type II abietadiene synthase from *Abies grandis*, while substituting a conserved histidine residue with aspartate opposite the DXDD motif, produced 8-hydroxy-CPP instead of CPP [86].

Pathway 1 shows the mechanism of Type I DTS, converting GGPP to diterpenoids. Pathway 2 shows the mechanism of Type II DTS. The representative cartoon structures of three different types of DTS are shown: monofunctional Type II DTS is AtCPS (PDB ID: 4LIX); monofunctional Type I DTS is TbTS (PDB ID: 3P5P); bifunctional Type I/II DTS is CPS/KS from *Physcomitrium patens,* whose structure is predicted by AlphaFold. The α-, β- and γ-domains of the DTS are indicated as well. The active sites of the DTS are shown as surfaces in magenta. GGPP: geranylgeranyl pyrophosphate. CPP: copalyl/labdadienyl diphosphate. TS: taxadiene synthase. 5αCYP: taxadiene-5α-hydroxylase. CPS: copalyl diphosphate synthase. KS: kaurene synthase. KO: kaurene oxidase. LS: levopimaradiene synthase. LPS: labdadienyl diphosphate synthase. SS: sclareol synthase.

### 2.2 Cytochrome P450 oxygenases (CYPs or P450s)

More extensive oxidation of diterpene scaffolds is typically performed by CYPs, significantly expanding the structural diversity of diterpenoids and providing a possibility for further functionalization. Many CYPs are non-specific and can transform a variety of analogous substrates via single-step hydroxylation, epoxidation, sequential oxidation, etc. [77]. A recent review extensively described the CYPs involved in plant diterpenoid metabolism [77]. Here, we want to highlight the different mode of oxidation for some CYPs depending on substrate. Kaurene oxidase (CYP701A3) from *Arabidopsis*, the multifunctional CYP that catalyzes the three-step sequential oxidation at the C_19_ position of *ent*-kaurene, changes to a single-step hydroxylase with *ent*-beyerene, which differs in the conformation of the 5-member ring [87]. Moreover, the taxadiene-5α-hydroxylase (5αCYP or CYP725A4) is an epoxidase when taxa-4(5)-11(12)-diene, the native substrate, is reacted. The epoxide intermediate decomposes spontaneously to form multiple products, including the desirable taxadiene-5-ol, rendering a pathway difficult to be engineered. In comparison, 5αCYP becomes a single-step hydroxylase when reacting with taxa-4(20)-11(12)-diene or iso-taxadiene, directly forming taxadiene-5-ol [88]. This led to TaS engineering to produce iso-taxadiene as an alternative intermediate to control the product specificity of 5αCYP [89]. These studies open a possibility to engineer product specificity of diterpenoids through CYPs.

### 2.3 Glycosyltransferases and acyltransferases

In addition to CYPs, GTs and ACTs also play important roles in diversifying diterpene structures [60]. GTs glycosylate diterpenes, greatly improving their solubility and stability, e.g., neoandrographolide and steviosides [90], [91], [92]. ACTs introduce acyl/aroyl groups into diterpenes. There are two families of ACTs, BAHD and serine carboxypeptidase-like (SCPL) ACTs [93]. BAHD ACTs are named from the first letter of each of the first four biochemically characterized enzymes of this family (BEAT, AHCT, HCBT, and DAT). They are further sub-classified into five clades, class V ACTs are involved in paclitaxel and forskolin biosynthesis [93] and a class III ACT (CfACT1-8) from *Coleus forskohlii* catalyzes the production of forskolin [94].

Aside from CYPs, GTs and ACTs, other unclassified enzymes can modify diterpenes by introducing ring-opening, rearrangement, carbon reduction, and polymerization, but are not covered in this review.

## 3 Sustainable production by metabolic engineering

Despite their broad applications, diterpenes are present in extremely low amounts in their original hosts. Plants are the major sources of natural diterpenes. However, plant growth requires not only massive land and water resources but is also susceptible to climate conditions, making diterpene production costly and difficult. In addition, the *de novo* chemical synthesis of diterpenes suffers from low yields and high cost due to the high complexity of diterpene structures.

Alternatively, microbial cell factories, powered by metabolic engineering and synthetic biology, are promising avenues to sustainably produce diterpenes. Of note, one- and two-step biotransformation of diterpenes are not covered in this review. Moreover, recently, plants such as the tobacco plant, *Nicotiana benthamiana,* have been metabolically engineered to produce terpenes [95]. We have not covered plant engineering here as our focus is the total biosynthesis of diterpenes using microbes from simple molecules such as sugars and glycerol.

To date, *Escherichia coli* and *Saccharomyces cerevisiae* are the two dominant workhorses due to advantages such as their well-studied genetics, being generally recognized as safe, their fast growth rate, the easy and reliable genetic manipulation toolboxes, and low culture costs [96,97]. In addition, *Yarrowia lipolytica*, a non-conventional yeast, is another promising chassis host for diterpene biosynthesis with advantages such as the inherent mevalonate pathway, high supply of acetyl-CoA and NADPH, and the naturally hydrophobic microenvironment [98]. Further, *Rhodosporidium toruloides* is another promising microbial workhorse, capable of supplying elevated amounts of acetyl-CoA, producing naturally diverse high-value compounds using a wide spectrum of carbon and nitrogen sources, and tolerant to inhibitors from lignocellulosic hydrolysates [99,100]. In addition, to produce native fungal diterpenes, the filamentous fungi *Aspergillus oryzae* and *Fusarium fujikuroi* have several advantages [101,102]. Last, photosynthetic algae such as *Chlamydomonas reinhardtii* [103] are potential microbial hosts for diterpene production.

**Fig. 3:**
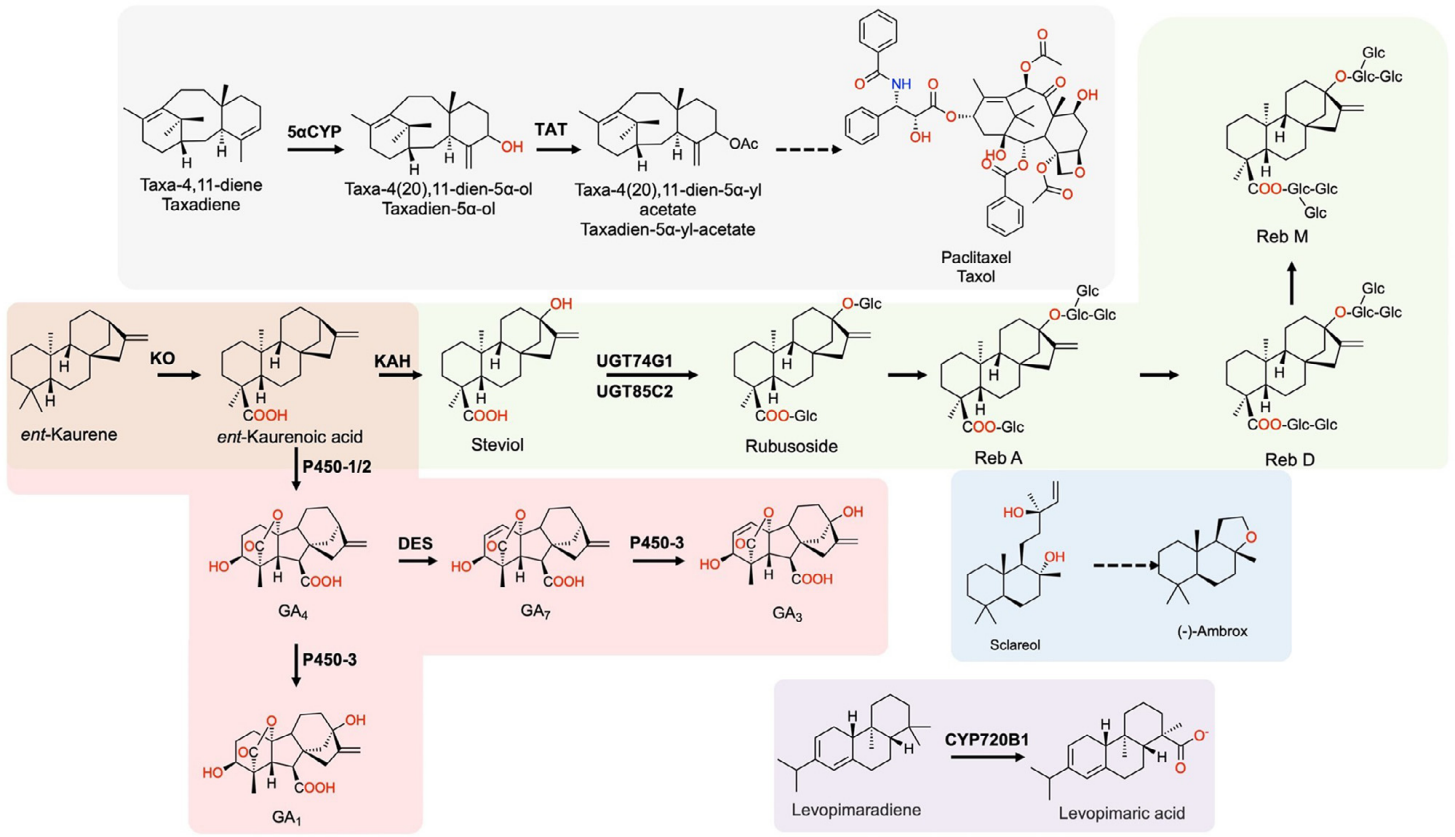
Biosynthesis pathways of selected high-value diterpenes and derived products.

Here, we review recent metabolic engineering efforts in the biosynthesis of diterpenes, especially those of high commercial value and large market size. We limited the scope of applications to agriculture, food, medicine, and personal care industries. Accordingly, we shortlisted taxanes, GAs, steviol glucosides, and sclareol (Fig. 3 and Table 2), as well as several other high-value diterpenes.

5αCYP, taxadiene-5α-hydroxylase; TAT, taxadien‐5‐alpha‐olO‐acetyltransferase; KO, kaurene oxidase; KAH, kaurenoic acid 13α-hydroxylase; UGT74G1, UDP-glycosyltransferase 74G1; UGT85C2, UDP-glycosyltransferase 85C2; DES, gibberellin desaturase; P450-1/2/3, three cytochrome P450 oxygenases from *F. fujikuroi* for gibberellin biosynthesis; CYP720B1, cytochrome P450 oxygenase 720B1.

**Table 2:** Summary of biosynthesis of diterpenoids in *E. coli* and fungi.

No.	Host	Diterpenoid products	Applications	Titre (mg/L)	Culturing conditions	During (hours)	Refs.
1	*E. coli*	Taxadiene	Medicine, the precursor to Taxol	1020	In a bioreactor, fed-batch	60	[104]
2	*S. cerevisiae*	Taxadiene	Medicine, the precursor to Taxol	20	In 5 ml YP-galactose medium	48-72	[106]
3	*S. cerevisiae*	Taxadiene	Medicine, the precursor to Taxol	129	In flasks, batch fermentation	72	[107]
4	*S. cerevisiae*	Oxygenated taxane	Medicine, the precursors to Taxol	78	In a bioreactor, fed-batch	120	[108]
5	*S. cerevisiae*	Taxadien‐5α‐yl‐acetate	Medicine, a precursor to Taxol	3.7	In a bioreactor, fed-batch	120	[108]
6	*E. coli and S. cerevisiae*	Taxadien-5α-acetate-10β-ol	Medicine, a precursor to Taxol	1	In a bioreactor, fed-batch	84	[109]
7	*FusariumMontliforme*	GA_3_	Agriculture	15910	In a bioreactor, fed-batch	240	[112]
8	*E. coli*	*ent*-Kaurene	Agriculture, food and medicine	624	In flasks, batch fermentation	24	[113]
9	*E. coli*	Steviol	Food and medicine	38.4	In a batch bioreactor	24	[113]
10	*Y. lipolytica*	GA_3_	Agriculture	12.8	in 24-roundwell plates	72	[114]
11	*Y. lipolytica*	GA_4_	Agriculture	17.3	in 24-roundwell plates	72	[114]
12	*R. toruloides*	*ent*-Kaurene	Agriculture, food and medicine	1400	In a batch bioreactor	281	[115]
13	*S. cerevisiae*	Rubusoside	Food and medicine	1369	In a bioreactor, fed-batch	120	[117]
14	*S. cerevisiae*	Rebaudiosides	Food and medicine	132.7	In a bioreactor, fed-batch	150	[117]
15	*E. coli*	Sclareol	Cosmetics, the precursor to Ambrox	1500	In a bioreactor, fed-batch	60	[119]
16	*S. cerevisiae*	Sclareol	Cosmetics, the precursor to Ambrox	403	In flasks, batch fermentation	60	[150]
17	*S. cerevisiae*	Sclareol	Cosmetics, the precursor to Ambrox	750	In flasks, batch fermentation	60	[121]
18	*E. coli*	Levopimaradiene	Medicine and an industrial intermediate used in coatings, printing inks etc	700	In a bioreactor, fed-batch	169	[141]
19	*S. cerevisiae*	Levopimaric acid	Medicine and an industrial intermediate used in coatings, printing inks etc	400	In a bioreactor, fed-batch	120	[142]
20	*E. coli*	Retinoids	Food, medicine and personal care	33	In tubes, batch fermentation	48	[259]
21	*S. cerevisiae*	Retinol	Food, medicine and personal care	2349	In a bioreactor, fed-batch	120	[260]
22	*Y. lipolytica*	Retinol	Food, medicine and personal care	4860	In a bioreactor, fed-batch	104	[159]
23	*S. cerevisiae*	Retinoic acid	Food, medicine and personal care	545	In a bioreactor, fed-batch	120	[261]
24	*A. oryzae*	Erinacines	Medicine	54.8	In flasks, batch fermentation	72	[144]
25	*S. cerevisiae*	Carnosic acid	Medicine and cosmetics	75.2	In a bioreactor, fed-batch	170	[140]
26	*A. oryzae*	Pleuromutillin and intermediates	Medicine as antibiotics	5-18	In CMP medium, batch fermentation	240	[124]
27	*S. cerevisiae*	Miltiradiene	Medicine, a key precursor to several drugs	3500	In a bioreactor, fed-batch	180	[131]
28	*S. cerevisiae*	Carnosic acid	Medicine	75.2	In a bioreactor, fed-batch	95	[140]

### 3.1 Taxanes

Taxol is a widely administered chemotherapeutic medicine to treat cancers. To date, the biosynthetic pathway of Taxol in its host, *Taxus chinensis*, is not fully understood. Hence, the heterologous biosynthesis of Taxol is incomplete; only several precursors can be produced in microbes. Taxadiene, the precursor to Taxol, has been produced in both *E. coli* and yeasts with the taxadiene synthase (TaS). In 2010, through a multivariate-modular approach, the upstream (*dxs, idi, ispD*, and *ispF*) and downstream (*TaS* and *GGPPS*) modules of the MEP pathway were elegantly balanced and the resulting engineered *E. coli* strain produced 0.3 in a shake flask and 1.0 g/L taxadiene in a 1-L bioreactor [104]. The strain was further engineered to produce 58 mg/L taxadien-5α-ol by introducing a fusion chimera protein of truncated 5αCYP and the cytochrome P450 oxidoreductase (CPR) redox partner from the Taxus species [104]. Considering the difficulties in expressing CYPs in *E. coli, S. cerevisiae* has been used to produce taxadiene. In 2008, *S. cerevisiae* was first engineered to produce 8.7 mg/L of taxadiene by overexpressing the tHMGR and introducing a *Sulfolobus* GGPPS to enhance the GGPP supply [105]. With a Cas9-based toolkit, the taxadiene production in *S. cerevisiae* improved by using 10 protein tags and five promoters of different strengths. The addition of the maltose binding protein solubility tag increased taxadiene production by 15 fold to 20 mg/L [106]. Using this solubility tag method, the taxadiene production increased to 129 mg/L by chromosomal integration and cultivation at a reduced temperature of 20°C instead of the commonly used 30°C [107]. Based on the same taxadiene engineered strain, the three genes, *T. cuspidata* CYP725A4, CPR, and taxadien‐5‐alpha‐ol O‐acetyltransferase (TAT), were introduced into the yeast genome (Fig. 3). Together, they convert taxadiene to taxadiene-5α-ol and further to taxadien‐5α‐yl‐acetate. Bioprocess optimization of oxygen and pH in micro and mini-bioreactors, allowed reaching titers of total oxygenated taxane and taxadien‐5α‐yl‐acetate of 78 mg/L and 3.7 mg/L, respectively (Table 2) [108]. Alternatively, a microbial consortium of *E. coli* and *S. cerevisiae* could produce up to 30 mg/L oxygenated taxanes, including 1 mg/L taxadien-5α-acetate-10β-ol [109]. This consortium system was stabilized by an artificially designed mutualistic relationship of the two species.

### 3.2 Gibberellins

Bioactive GAs, a subclass of diterpenoids, are plant hormones that regulate plant development and growth including seed germination, stem elongation, leaf expansion, flower induction, and seed growth [110]. To date, > 100 GAs have been identified in plants, among which GA_1_, GA_3_, GA_4_, and GA_7_ are the major bioactive ones [110]. According to their wide applications in crops and fruits production, the global GA market is projected to reach US$ 1.42 billion by 2027 [https://www.bloomberg.com/press-releases/2020-06-23/global-gibberellins-market-to-reach-usd-1-42-billion-by-2027-reports-and-data]. In addition to plants, the rice pathogenic fungus *Gibberella fujikuroi* (mating population C) can produce large amounts of GAs, especially GA_3_ and its precursors, GA_4_ and GA_7_ (Fig. 3) [111]. Several fermentation optimization strategies have been applied to increase GA_3_ production with this fungus. Among them, nitrogen limitation, fed-batch glucose feeding, alternative carbon courses (e.g., sucrose, glycerol, maltose, starch, and plant oils) are very effective. In addition, maintaining a high oxygen supply is important in GA_3_ production. Further optimizing pH and temperature, the GA_3_ titer reached 15.9 g/L in optimized submerged fermentation using a *Fusarium Montliforme* strain [112] (Table 2). With the development of genetic modification tools, *Fusarium fujikuroi* was engineered to overproduce GA_4/7_ by disrupting P450-3 and overexpressing tHMGR and CPS/KS. The engineered strain produced 292.5 mg/L GA_4_ and 423.9 mg/L GA_7_ [102].

In addition to native producers, non-native hosts such as *E. coli* [113], *Y. lipolytica* [114], and *R. toruloides* [115] have been engineered to produce GAs and its precursors, *ent*-kaurene and *ent*-kaurenoic acid. To increase the supply of terpene precursors, the genes *dxs, idi, ispA*, and *dxr* were overexpressed in *E. coli* genome. Moreover, the genes GGPPS, *ent*-CPS, and KS were further knocked into the *E. coli* genome. Together with 5’UTR optimization, the strain produced 623.6 mg/L of *ent*-kaurene. Further, the introduction of truncated kaurene oxidase (AtKO) and CPR (named AtCPR2) from *Arabidopsis thaliana* enabled the strain to produce 50.7 mg/L *ent*-kaurenoic acid [113]. Similarly, in *Y. lipolytica*, the terpene pathway was first enhanced by overexpressing tHMGR and GGPPS and down regulating squalene synthase. Further overexpression of AtKO, NADPH-dependent CYP (AtATR2), and *Y. lipolytica* cytochrome b5 (YlCyb5) enabled the strain to produce 3.8 mg/L kaurenoic acid. Further, this strain was modified to overexpress the *Synechococcus sp.* GGPPS (SsGGPPS7) and three CPR redox partners, cytochrome b5, cytochrome b5 reductase, and cytochrome P450 reductase (GfCyb5, GfCyb5Red, GfCPR) from *Fusarium fujikuroi*. With this strain, different combinations of P450 enzymes were introduced to produce different GAs: GfP450-1 and GfP450-2 to produce GA_4_; GfP450-1, GfP450-2, GfP450-3, and GA desaturase to produce GA_3_. Finally, of the various strains obtained, the best ones produced 17.3 mg/L GA_4_ or 12.8 mg/L GA_3_ [114] (Table 2). The kaurene production in *R. toruloides* increased by overexpression of a mutant of farnesyl diphosphate synthase (FPPS) from *Gallus gallus* (GgFPS-F112A) and KS. This engineered red yeast produced 1.44 g/L kaurene from the lignocellulosic biomass of a corn stover hydrolysate comprising ∼75 g/L glucose and ∼40 g/L xylose [115].

### 3.3 Steviol glycosides

High sugar uptake is a global concern leading to several chronic diseases such as obesity, diabetes, and metabolic syndrome. Singapore's Ministry of Health has introduced measures to reduce the daily sugar uptake to fight against diabetes since 2016. Steviol glycosides are one of the most popular natural sweeteners, with potent sweetness (∼300 fold that of sucrose) and very low calorie content [116]. Steviol and GAs share several common precursors in the biosynthetic routes and diverge from *ent*-kaurenoic acid. *Ent*-kaurenoic acid oxidase (KAO) or GfP450-1 catalyzes *ent*-kaurenoic acid to GA12. Kaurenoic acid 13-hydroxylase (KAH) converts *ent*-kaurenoic acid to steviol and then to rubusoside by two UDP-dependent glycosyltransferases (UGTs), UGT74G1 and UGT85C2. The glycosylation step of steviol is versatile, leading to various types of derivatives (e.g., glucosyl, rhamnosyl, fucosal and xylosyl) and different numbers of sugar residues. Among different steviol glucosides, rebaudioside A (Reb A), rebaudioside D (Reb D), and rebaudioside M (Reb M) (Fig. 3) are particularly interesting as they have the highest sweetness intensities. In addition, Reb D and Reb M do not have a bitter aftertaste, an attractive feature for food and beverage applications [117]. The *E. coli* designed for *ent*-kaurenoic acid-production has been further engineered to produce 38.4 mg/L steviol by introducing the fusion protein UtrCYP714A2-AtCPR2 derived from *A. thaliana* [113]. Recently, a modular approach has been applied to engineer *S. cerevisiae* which can produce rubusoside and rebaudiosides (a mixture of Reb A, D, and M) in a sequential optimization strategy. The yeast strain was first engineered to produce kaurene by overexpressing KS, tHMGR, IDI, and the mutant yeast FPPS-F112A. The two P450s, KAO from *S. rebaudiana* and KAH from *A. thaliana*, and one cytochrome P450s reductase were introduced in the optimized kaurene strain to produce steviol. Subsequently, UGT74G1 and UGT85C2 from *S. rebaudiana* were integrated in the genome of the steviol strain to produce rubusoside. This enzyme fusion strategy and the use of efflux transporters greatly increased the rubusoside yield (Fig. 3). The optimized strain produced 1368.6 mg/L in fed-batch fermentation (Table 2). Next, this rubusoside strain was engineered to produce the three rebaudiosides (Reb A, D and M) with the combined expression of several UGTs. The optimized strain produced 21.5, 44.2, and 67.0 mg/L of Reb A, Reb D, and Reb M, respectively, in a fed-batch bioreactor [117].

### 3.4 Sclareol

Bacteria and yeasts have been explored to produce sclareol, an important fragrance molecule, and key precursor to synthesize (−)-Ambrox (Fig. 3), a valuable perfume fixative due to its unique scent [118]. The Firmenich team pioneered the sclareol biosynthesis in *E. coli*. The sclareol biosynthetic pathway from *Salvia sclarea* was elucidated and subsequently re-constituted in *E. coli* through cloning and functional characterization. The optimized strain produced 1.5 g/L in bioreactors [119] (Table 2). Later, a *S. cerevisiae* strain was engineered to produce sclareol, as well as two other diterpenes, *cis*-abienol and abietadiene [120]. In this process, GGPP synthesis was the main bottleneck for diterpenoid production. The GGPPS from *Cistus criticus* was introduced to supplement the insufficient activity of GGPPS (*BTS1* gene) in yeasts. Moreover, a F96C mutation was introduced to the yeast Erg20 (FPP synthase), enhancing the enzymatic activity for GGPP production. Together, the sclareol production increased to 403 mg/L in flasks. Another study applied iterative carotenogenic screens (a colorimetric method) to identify the targets for gene deletion. Through several iterative screenings, a sextuple-gene mutant (*rox1, dos2, yer134c, vba5, ynr063w* and *ygr259c*) that produced 750 mg/L sclareol in shake flasks was identified [121] (Table 2). Sclareol production was also tested using the green algae *C. reinhardtii*; the bottlenecks of the MEP pathway were identified and overcome by enzymatic fusion [103]. Photoautotrophic high-cell-density fermentation combined with metabolic engineering resulted in a yield of 656 mg/L sclareol at day 19 in a 2.5 L illuminated cultivator. This demonstrates the promise of microalgal diterpenoid production.

### 3.5 Other valuable diterpenoids

Pleuromutilin and its derivatives, such as lefamulin and valnemulin, are important antimicrobial diterpenes widely used as antibiotics in humans and other animals [122]. Its biosynthetic gene cluster in *Clitopilus passeckerianus* was identified [123]. Heterologous pleuromutilin production was achieved by reconstruction of the entire 7-gene cluster in *A. oryzae* [123]. Furthermore, the genes in the cluster were expressed stepwise and the structures of the intermediates were elucidated. A mutant library of *A. oryzae* was constructed to produce various intermediates along the pleuromutilin biosynthetic pathway as final products with a titer range between 5 and 18 mg/L [124] (Table 2).

Conidiogenone was first isolated from *Penicillium cyclopium* as a conidiogenesis inducer and can induce conidiation in *P. cyclopium* [125]. Its biosynthetic pathway was functionally identified by heterologous expression in *A. oryzae* [126]. However, sustainable production of conidiogenone and its derivatives in microbes has not been achieved, despite the success of total chemical synthesis [127,128].

Miltiradiene is a key intermediate in a series of pharmaceutically important diterpenes, including triptolide, tanshinones, carnosic acid, and carnosol [129]. A modular pathway engineering strategy was applied for rapid assembling of synthetic miltiradiene pathways in *S. cerevisiae*. The fusion of miltiradiene synthase and its upstream enzymes increased miltiradiene production to 365 mg/L in a 15-L bioreactor [130]. Hu et al. further improved the yield to 3.5 g/L in a 5-L bioreactor by coupling geranylgeraniol biosynthesis with the overexpression of chimeric DTSs from *Coleus forskohlii* and *Salvia miltiorrhiza* [131] (Table 2).

Carnosic acid (CA) is an abstracting phenolic tricyclic diterpene [132] characterized by various biological effects and used in pharmaceuticals, cosmetics, food additives, and spices [133], [134], [135], [136]. The first engineering trial produced 1 mg/L CA by fusing the FPP synthase mutant Erg20p(F96C) with CPS and overexpressing the hydroxymethylbilane synthase gene (HEM3) [137]. A subsequent trial produced 2.7 mg/L CA by expressing GGPPS, miltiradiene synthase, CPS, P450 reductase (ATR1), and the two P450s (CYP76AH1 and CYP76AK8) [138]. Modifying the first trial by adjusting the linker length of the fusion protein and balancing the co-expression of CPR, P450s, and cytochrome b5 (Cytb5) resulted in 18 mg/L CA [139]. Recently, CA production reached 24.7 mg/L in shaking flask culture and 75.2 mg/L in a 5-L fed-batch bioreactor (Table 2) by 1) expressing a CPS and a kaurene synthase-like enzyme for miltiradiene synthesis, 2) integrating the genes encoding P450s and CPR (SmCPR) for construction of CA's biosynthetic pathway, 3) co-expressing the P450 (CYP76AH1) and SmCPR∼t28SpCytb5 fusion protein, 4) expressing various catalases to detoxify the hydrogen peroxide generated, and 5) engineering the endoplasmic reticulum and cofactor supply [140].

Levopimaradiene is a precursor to pharmaceutically important ginkgolides and an important industrial intermediate of products used in coatings, plasticizers, and adhesive and printing inks [141,142]. Its production in *E. coli* improved by protein engineering of GGPPS and levopimaradiene synthase (LPS) through combinatorial mutations [141]. The optimized strain produced 700 mg/L levopimaradiene in fed-batch fermentation. Next, levopimaric acid (Fig. 3), a diterpene resin first isolated from conifers, was produced in yeast by introducing LPS, CYP720B1, and cytochrome P450 reductases (CPR) from *Taxus cuspidate*. Through N-terminal truncation and site-directed mutagenesis of LPS and multi-copy δ site integration of CYP720B1 and CPR, the levopimaric acid titer reached 400 mg/L via fed-batch fermentation in a 5-L bioreactor [142] (Table 2).

Cyathanes are a group of mushroom-derived natural tricyclic diterpenes with an angularly fused 5/6/7 tricyclic skeleton [4]. Erinacines, first isolated from *Hericium erinaceus*, are a group of cyathane-type diterpenes with a rare structure of cyclohepta-1,3-diene, which could be a potential treatment for neurodegenerative diseases [143]. Investigation of an *A. oryzae* mutant library using whole-genome sequencing revealed several high expression loci (hot spots) in which the integrated genes showed higher expression levels [144]. Based on this technique, the erinacine biosynthetic gene cluster from *H. erinaceus* was reconstructed into expression hot spots; the resulting strain produced 30.6 mg/L of erinacine Q [144] (Table 2).

In summary, the number of diterpenes produced by engineered microorganisms is increasing rapidly. The achieved titers have gradually increased from “mg/L” to “g/L” levels for taxadiene, sclareol, GA_3_, retinol, rebucoside, *ent*-kaurene, and miltiradiene as summarized in Table 2. Typically, a 1 g/L titer is a good starting point for further optimization toward commercialization. However, the general yields of diterpenes are relatively lower compared to that of sesquiterpenes, 20–100 g/L [145,146] which are or have been commercialized. This is partly due to the structural complexity of diterpenes and the low activities of key enzymes such as CYPs, which limit the efficiency of diterpene biosynthesis.

### 3.6 Engineering strategies

The great achievements on microbial synthesis of diterpenes and many other natural products share several common strategies [58,98,147]. Here, we briefly summarize those applied to terpene biosynthesis. First, key enzyme overexpression and blocking or down regulating competing pathways are basic but effective metabolic engineering strategies. For example, almost all studies in yeasts using the MVA pathway overexpressed tHMGR and GGPPS for diterpene biosynthesis. For the MEP pathway, DXS*,* IDI, and GGPPS overexpression is very common [104,148]. As gene deletion strategy, sclareol biosynthesis is a good example [121]. Second, modular pathway engineering strategies are very useful especially for prokaryotes, in which multiple genes can be arranged under the regulation of the same promoter. There are three subgroups of strategies using modular metabolic engineering: (1) simple modular engineering, which builds and tests all combinations, such as the biosynthesis of taxadiene [104], steviol glucosides [117], and miltiradiene [130]; (2) that facilitated by experimental design and regression models, such as amorphadiene synthesis, which simultaneously balanced four modules with experimental design [148]; (3) a highly flexible multidimensional heuristic process that can regulate both inter- and intra-modules by predefined regulatory elements and a knowledge-learning cycle by reverse engineering [149]. Third, enzyme engineering is critical for the biosynthesis of diterpenoids, especially for P450s, which have low expression, poor activity, and require CPR redox partners. Common enzyme engineering strategies including N-terminal modification (truncation, fusion partners, membrane anchoring, etc.), fusion CYP and CPR, direct evolution, and structure-guided enzyme engineering. Examples are found in the biosynthesis of taxanes, sclareol, and steviol glucosides [104,117,150]. Fourth, other emerging strategies such as compartmentalization in mitochondria, peroxisome, lipid droplet, and endoplasmic reticulum, and efflux transporter engineering are reported elsewhere [151].

## 4 Conclusions and future perspectives

Currently, commercial diterpenes are dominantly produced by plant extraction and chemical synthesis. However, the unsustainability and inefficiency of both processes highly restrict their expansion and application. Considering the similarities in biosynthetic pathway and enzymes, the microbial synthesis of diterpenes is advantageous for various diterpenes and promising with respect to its sustainability, high efficiency, enantiopurity, and expandability.

The technological revolution led by next generation sequencing, omics analysis, synthetic biology, and metabolic engineering, has greatly increased our knowledge of genes, enzymes, and pathways involved in the synthesis of industrially relevant and novel diterpenes. This provides extraordinary opportunities to boost their yields in heterologous microbial hosts in a sustainable and efficient production. As summarized in this review, over 20 high-value diterpenes have been successfully produced in chassis microbes or using the native hosts, in some cases achieving >1 g/L titers, which are close to commercialization.

To further expand the diterpene products that can be biosynthesized in microbes and build more efficient and robust microbial cell factories, we propose the following strategies: (1) Developing more efficient multi-segment and long-segment DNA assembly technologies and genome editing methods; (2) comprehensively understanding the metabolic network and regulatory mechanisms of natural producers and chassis microbes through in-depth omics research; (3) developing high-throughput assays for rapid detection of intracellular products and intermediate metabolites; (4) diagnosing and improving the activities of key enzymes by enzyme engineering such as directed evolution, truncation, and enzyme fusion; (5) adopting modular engineering strategies [152,153] for pathway design, optimization, and progressive production of diterpene intermediates and final products; (6) identifying and maximizing the storage and transport for diterpenes in chassis cells by subcellular localization, compartmentalized regulation, and efflux transporters [151,154]; and (7) shortening the design-build-test-learn cycle of cell factories by building automated and intelligent synthetic biology platforms [155].

## Declaration of Competing Interest

The authors declare that they have no known competing financial interests or personal relationships that could have appeared to influence the work reported in this paper.
